# High-resolution particle separation by inertial focusing in high aspect ratio curved microfluidics

**DOI:** 10.1038/s41598-021-93177-w

**Published:** 2021-07-06

**Authors:** Javier Cruz, Klas Hjort

**Affiliations:** Division of Microsystems Technology, Uppsala University, Ångström Laboratory, Uppsala, Sweden

**Keywords:** Biosensors, Microfluidics, Nanoparticles

## Abstract

The ability to focus, separate and concentrate specific targets in a fluid is essential for the analysis of complex samples such as biological fluids, where a myriad of different particles may be present. Inertial focusing is a very promising technology for such tasks, and specially a recently presented variant, inertial focusing in High Aspect Ratio Curved systems (HARC systems), where the systems are easily engineered and focus the targets together in a stable position over a wide range of particle sizes and flow rates. However, although convenient for laser interrogation and concentration, by focusing all particles together, HARC systems lose an essential feature of inertial focusing: the possibility of particle separation by size. Within this work, we report that HARC systems not only do have the capacity to separate particles but can do so with extremely high resolution, which we demonstrate for particles with a size difference down to 80 nm. In addition to the concept for particle separation, a model considering the main flow, the secondary flow and a simplified expression for the lift force in HARC microchannels was developed and proven accurate for the prediction of the performance of the systems. The concept was also demonstrated experimentally with three different sub-micron particles (0.79, 0.92 and 1.0 µm in diameter) in silicon-glass microchannels, where the resolution in the separation could be modulated by the radius of the channel. With the capacity to focus sub-micron particles and to separate them with high resolution, we believe that inertial focusing in HARC systems is a technology with the potential to facilitate the analysis of complex fluid samples containing bioparticles like bacteria, viruses or eukaryotic organelles.

## Introduction

Benefitting from the features of miniaturized systems, microfluidics have key features such as the need of small volumes of samples and reagents, fast responses, high sensitivity, portability, and low cost^1,2^. Within microfluidics, inertial focusing provides the means for a precise manipulation of particles, allowing for tasks such as focusing, separation and concentration of targets of interest within a fluid where a myriad of other particles may be present^3–5^. Such processing has been shown to facilitate the detection and extraction of rare targets of interest from fluid samples such as circulating tumour cells from blood^6–8^ or microalgae from water^9,10^. Manipulation of particles in microfluidics, however, becomes more challenging as smaller particles are targeted because the forces in play strongly depend on the particle size^11,12^. Wang et al. recently reviewed the state of the art of different microfluidic technologies that allow focusing of sub-micron particles^11^, with viscoelastic^13,14^ and DLD^15–17^ microfluidics showing promising results in such range. As for inertial focusing, recent advances have also shown focusing of particles in the sub-micron range, including polystyrene particles and bacteria in water^18,19^, with the smallest focused size reported being 0.5 µm. Noteworthy is the innovative approach taken by Mutlu et al., presented as *Oscillatory inertial focusing in infinite microchannels,* that also allowed focusing of such small sizes^20^.

**Figure 1:**
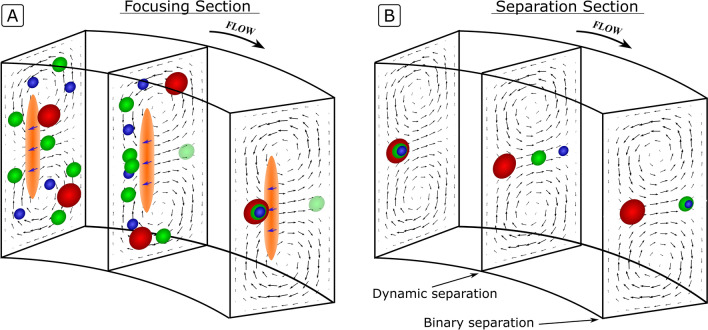
(**A**) 3D focusing mechanism in HARC systems. The Lift Barrier (orange ribbon) prevents particles from crossing to the outer wall, resulting in a close focus position for all sizes. (**B**) Particle separation in HARC systems. With all particles starting at a similar position, the Lift Barrier is weakened, allowing the smaller ones to migrate towards the outer wall.

The separation capabilities of such systems are, however, limited, because the focus positions not only depend on the particle size but also shift in a complex manner as a function of the channel geometry and flow rate^4,21–23^. For most geometries, this makes the systems exploiting inertial focusing impractical and difficult to engineer. Recently, we reported that inertial focusing in High Aspect Ratio Curved (HARC) systems overcomes this limitation by providing a single focus position, common for a large range of particle sizes and practically invariant with the flow rate^24,25^ (Fig. 1A). However, while convenient for focusing and fractionating a range of sizes together, as presented, HARC systems lost the capacity to separate the particles by size; a key feature of inertial focusing.

Within this paper, we recuperate this lost feature and introduce the use of HARC systems for high-resolution separation of particles with different sizes. The key advantage compared to other systems is that in HARC systems the particle positions do not shift in tortuous manners. Instead, all particles are pre-focused together at the inner wall and, when they enter the separation section, they migrate with a size-dependent speed towards the outer wall, achieving a predictable and modulable separation. We present the idea, a theoretical model, and demonstrate it by the separation of 0.79, 0.92 and 1.0 µm polystyrene particles in silicon-glass microchannels. The model matched well the experiments, and the resolution of the separation could be modulated with the radius of curvature of the channel.

### Introduction to HARC systems

A brief summary about inertial focusing in HARC systems is included in the following paragraphs to ease the understanding of the proposed mechanism for particle separation.

A curved system can be approximated as an overlap of a straight channel, where a lift force ($F_{L}$) arises and pushes particles from the centre towards the walls^26,27^, and the secondary flow induced by the curvature, where two vortexes appear and make particles circulate following their orbits^28,29^. In the particular case of the HARC systems, where the microchannel geometry is defined by the channel width (*W*), the height (*H*), the aspect ratio ($AR = H/W > 1$) and the radius of curvature (*R*), *F*_*L*_ opposes the vortexes by the inner wall, and its horizontal component acts as a barrier (referred to as the Lift Barrier, *B*_*L*_) (Fig. 1A). Provided that the Lift Barrier is stronger than the horizontal drag of the vortexes (*F*_*Dx*_), particles cannot cross over to the outer wall and the vertical component of vortexes brings them to a single focus position at the symmetry line^24,25^. Given a sufficient channel length, all particles that were initially distributed over the cross section reach this focus position (Fig. 1A). This length, expressed as the number of loops of the microchannel (*N*_*L*_), can be estimated by Eq. (1)^24^:

1 $$N_{L} \approx \frac{{20(AR)^{2} }}{{Re}}$$

where *Re* is the Reynolds number of the channel, defined as $\text{Re} = \frac{{\rho U_{m} W}}{\mu }$, with $\rho$ and $\mu$ being the density and dynamic viscosity of the fluid and *U*_*m*_ the maximum flow velocity.

If, on the contrary, the vortexes are stronger, particles surpass the Lift Barrier and remain randomly distributed. The condition for particles not to cross the Lift Barrier, expressed as a fraction in Eq. (2) (force balance) ^25^:

2 $$\frac{{B_{L} }}{{F_{{Dx}} }} = \frac{J}{{3~\pi }}\frac{{a^{3} R}}{{C_{{ROI}} U_{m} W^{5} }} > 1$$

where $J = 3.6{\text{~}}\pi {\text{~}}10^{{ - 6}} \;\frac{{m^{2} }}{s}$ is the Lift Barrier constant, *a* is the diameter of the particle, *R* the radius of curvature of the microchannel, and ${\text{C}}_{{ROI}} = (6.55 - 1.87(AR)) \times 10^{{ - 3}}$.

With Eq. (2), defining the conditions for particles to stop at the inner wall, and Eq. (1), defining the channel length for particles to reach the focus position, HARC systems can be engineered to focus a desired range of particle sizes together. This is a prerequisite for our separation method in HARC systems. In the following paragraphs, we extend the use of HARC systems to also enable particle separation.

### Particle separation in HARC systems

Our separation method is based on engineering the Lift Barrier to selectively allow particles to cross over to the outer wall. This scenario lacks interest if particles are initially randomly distributed over the cross section, as they would remain in that way. However, if the particles are initially focused at a single position by the inner wall, modulating the barrier can be used for size-based separation as sketched in Fig. 1B.

As the force balance (Eq. 2) is sensitive to the particle size, the Lift Barrier acts as a threshold, retaining particles larger than a certain size while allowing the smaller ones to travel towards the outer wall. If the separation section is long enough for all released particles to reach the outer wall, a binary separation is achieved with two focus lines (larger and smaller particles than the threshold); *cf.* the binary state in Fig. 1B. This threshold can be precisely and continuously tuned by changing the variables in Eq. (2) (for instance, by changing *R*). Mathematically, it can take any value and therefore allow for the separation of particles with minute size differences; i.e., separation with unlimited resolution. In practice, the resolution has practical limits such as the quality of the fabrication, the stability of equipment to run the system, or the maximum pressure allowed in the system.

Interestingly, when the Lift Barrier is surpassed, *F*_*L*_ induces a lag to the particles that translates into a size-dependent migration velocity. As a result, when particles of different sizes are released simultaneously from a similar position, their trajectories form a rainbow of sizes during their migration towards the outer wall; cf. the dynamic state in Fig. 1B. This provides the means to separate particles over a size gradient, rather than in two groups as in the binary state. Note, however, that in this case particles are not in equilibrium but migrating and their position must be precisely predicted. Therefore, designing this kind of separation requires a deeper understanding than simply lowering the Lift Barrier and having a sufficient channel length, which is the scope of the following paragraphs.

## Theory

### Model for particle migration in microfluidics

**Figure 2:**
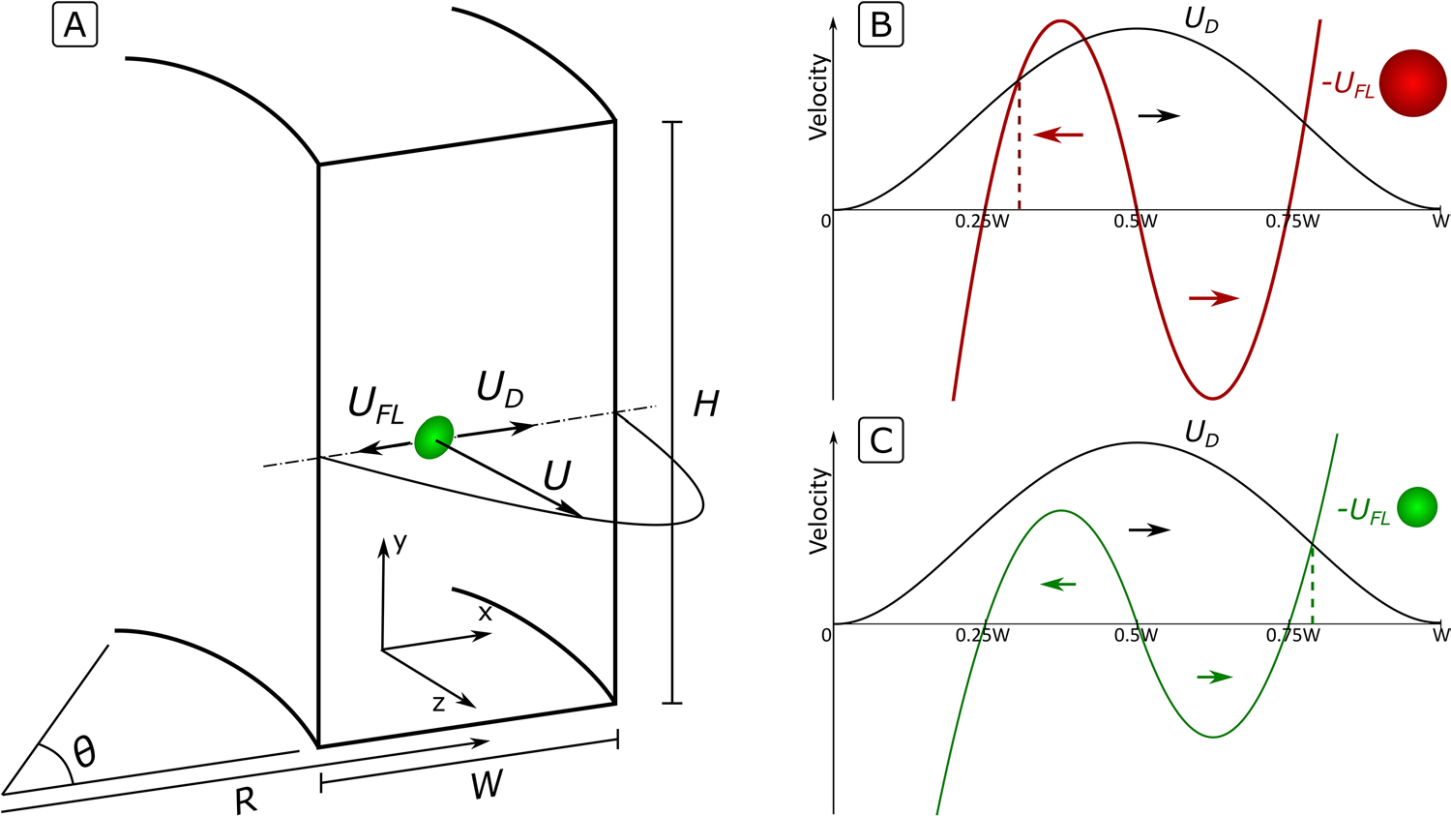
(**A**) Variables in a HARC system (**B**,**C**) Magnitude and direction of $U_{D}$ and $U_{{FL}}$ at the symmetry line for (**B**) a situation where $U_{{FL}}$ dominates; $U_{D}$ does not surpass the Lift Barrier and the particle remains at the inner wall (**C**) a situation where $U_{D}$ dominates; it surpasses the Lift Barrier and the particle travels towards the outer wall. The dashed lines indicate the focus position (provided the initial position is 0.25 $W$).

In the following paragraphs, we build a model to estimate the velocity of particles in microfluidic HARC channels and use it to describe their trajectories. A sketch of a HARC channel is depicted in Fig. 2A. The variables defining the microchannel geometry are the width (*W*), the height (*H*), the aspect ratio ($AR = H/W$), the radius of curvature (*R*) and the channel length expressed as the angle rotated ($\theta$). The main direction of the microchannel is in the axial direction Z and the transversal directions are X and Y for the width and height of the channel, respectively. The origin of coordinates (*x*, *y*) is set at the point where the symmetry line intersects the inner wall.

In the model, the velocity of the particles was assumed to be similar to that of freely suspended particles plus an induced component in response to a force (lift force, in this case). In microfluidics, the velocity of freely suspended particles can be approximated to that of the fluid at every position, even if the fluid changes speed (e.g., in a constriction or an expansion of the channel). This is derived from two facts:

(1) A drag force arises on a particle if this has a mismatch of velocity with respect to the fluid, making it accelerate until the relative velocity becomes null^30,31^.

(2) In systems where the particle Reynolds number is low, the acceleration time for particles to reach the equilibrium velocity is negligible, as beautifully explained by Purcell in 1977^32^. Please note that this assumption was made for a very low particle Reynolds number and in inertial focusing it may reach higher numbers. Nevertheless, the simplification in the calculations is noteworthy and, as the reader will see in the “Results” section, the model predicts the particle trajectories with accuracy.

The velocity of the fluid through a curved microchannel can be divided into a main flow (*U*), in the direction of the microchannel (its axial direction, Z) and a secondary flow (Dean flow, *U*_*D*_) orthogonal to the main flow (transversal direction) that is originated by the curvature of the channel (Fig. 2A). A freely suspended particle then moves with a velocity *U* in the axial direction and *U*_*D*_ in the transversal direction.

On the other hand, if the particle is not freely suspended but a force (*F*) acts on it, a relative velocity (*U*_*r*_) is induced with respect to the fluid. The velocity induced by *F* can be calculated by assuming it is countered by the drag from the fluid ($F_{D} = F$). At low $Re$, $F_{D}$ can be calculated as a Stokes drag; $F_{D} = 3\pi \mu aU_{r}$, and the force induces a relative velocity on a particle $U_{r} = \frac{F}{{3\pi \mu a}}$. In the case of inertial focusing, the dominant force is the lift force (*F*_*L*_)^3,22,26^, which induces a transversal velocity (*U*_*FL*_) on the particles:

3 $$U_{{FL}} = \frac{{F_{L} }}{{3\pi \mu a}}$$

The velocity of a particle in an inertial focusing system thus becomes *U* in the axial direction and $U_{D} + U_{{FL}}$ in the transversal direction (Fig. 2A).

Having the velocity of a particle described, its trajectory within the microchannel can be calculated. In an infinitesimal interval of time, the displacement in the axial direction of the microchannel is $dl = Udt$. In the same interval of time, the particle moves along the cross section $dx_{i} = (U_{{Di}} + U_{{FLi}} )dt$, with the subindex $i$ indicating the two possible Cartesian components $(x,\;~y)$, see Fig. 2A. Bringing both expressions together relates the axial and transversal displacements: $dx_{i} = \frac{{U_{{Di}} + U_{{FLi}} }}{U}dl$. Finally, expressing $dl$ as $dl = Rd\theta$, the axial displacement is related to the angle rotated in the curved channel:

4 $$dx_{i} = \frac{{U_{{Di}} }}{U}Rd\theta + \frac{{U_{{FLi}} }}{U}Rd\theta$$

The first term on the right side of Eq. (4) represents the displacement of a fluid molecule, and the second term the displacement induced on a particle by $F_{L}$. The integration of Eq. (4) provides the position of the particle in the cross section as a function of the angle rotated by the microchannel $(x,\;~y) = f(\theta )$.

In the following section, Eq. (4) is solved along the symmetry line, obtaining the trajectories of pre-focused particles in the process of separation in HARC systems.

### Solution at the symmetry line for HARC microchannels

Figure 2A shows a general case for $U$, $U_{D}$ and $U_{{FL}}$ at the symmetry line of a HARC microchannel, and Fig. 2B,C show the detailed profile of $U_{D}$ and $U_{{FL}}$. In the half of the channel closer to the inner wall, $U_{D}$ and $U_{{FL}}$ oppose each other. If $U_{{FL}}$ is larger than $U_{D}$ at any position, particles are not able to cross to the outer wall, Fig. 2B and 1B for the largest particle. However, when $U_{D}$ is superior to $U_{{FL}}$ at every position, particles cross over to the outer wall, Fig. 2C and 1B for the medium and small particles. In this case, $U_{{FL}}$ translates into a lag that is proportional to the size of the particle and, provided that all particles are initially focused at a single point, allows for separation by size.

Analytical expressions for $U$, $U_{D}$ and $U_{{FL}}$ were studied at the symmetry line to solve Eq. (4). The position along the symmetry line was normalized by $W$, making $x = 0$ and $x = 1$ the positions of inner and outer walls, respectively.

#### Analytical expression for *U*

The main flow at the symmetry line follows a parabolic distribution (Fig. 2A). Its value along the line can be mathematically expressed as:

5 $$U = U_{m} f_{U} \left( x \right)$$

with $U_{m}$ being the maximum value of the velocity and $f_{U} \left( x \right)$ a function that adjusts the value to the position.

Different flows were computed with COMSOL Multiphysics, obtaining $f_{U} \left( x \right) = U/U_{m} \approx 4\left( {x - x^{2} } \right)$.

#### Analytical expression for *U*_*D*_

At the symmetry line, the secondary flow moves from the inner wall towards the outer one (Fig. 2B,C). Its value along the line can be mathematically expressed as:

6 $$U_{D} = C_{{ROI}} \frac{\rho }{\mu }\frac{{U_{m}^{2} W^{2} }}{R}f_{{U_{D} }} (x)$$

with $C_{{ROI}} \frac{\rho }{\mu }\frac{{U_{m}^{2} W^{2} }}{R}$ expressing its magnitude and scaling^25^ and $f_{{U_{D} }} (x)$ being a function that adjusts the value to the position.

Different flows were computed with COMSOL Multiphysics, obtaining $f_{{U_{D} }} (x) = U_{D} /\left( {C_{{ROI}} \frac{\rho }{\mu }\frac{{U_{m}^{2} W^{2} }}{R}} \right) \approx (21.25x^{4} - 42.51x^{3} + 21.43x^{2} - 0.18x)$; see shape in Fig. 2B,C.

#### Analytical expression for *U*_*FL*_

In the particular case of inertial focusing in HARC microchannels, $F_{L}$ along the central line is comparable to that of a Poiseuille flow, first described analytically by Ho and Leal in 1974^26^. In this work, as a simplified but accurate model, we considered $F_{L}$ with a distribution like that proposed by Ho and Leal—two antisymmetric parabolas—with its strength defined as measured experimentally in our previous work^25^. Mathematically, $F_{L} = F_{{Lmax}} f_{{FL}} (x)$, with $F_{{Lmax}} = J\rho U_{m} a^{4} /W^{3}$ representing the magnitude^25^, and $f_{{FL}} (x)$ adjusting its strength along the central line and approximated to two antisymmetric parabolas, as shown in Fig. 2B,C. In the first half of the channel, $f_{{FL}} (x) = - \left( {1 - \frac{{(x - 0.375)^{2} }}{{(0.125)^{2} }}} \right)$, and in the second half $f_{{FL}} (x) = \left( {1 - \frac{{(x - 0.625)^{2} }}{{(0.125)^{2} }}} \right)$.

In this model, with such definition of the distribution,$~f_{{FL}} (x)$, we introduce an important simplification, as we assume that the distribution of the force is invariant with respect to the particle size and *Re* number. In the literature, however, small variations as function of particle size and *Re* have been reported^33,34^. Yet, as the reader will see in the section Results*,* the model explains the trajectories remarkably well.

Following Eq. (3), the velocity induced by *F*_*L*_ along the central line is:

7 $$U_{{FL}} = \frac{J}{{3\pi }}\frac{\rho }{\mu }U_{m} \frac{{a^{3} }}{{W^{3} }}f_{{FL}} (x)$$

#### Particle displacement

Equation (4) can be re-organized to reflect the relevance of $U_{{FL}}$ in the trajectories in relative terms:

8 $$dx = \frac{{U_{D} }}{U}Rd\theta \left( {1 + \frac{{U_{{FL}} }}{{U_{D} }}} \right)$$

Equation (8) indicates that the migration of a particle will be based on the fluid displacement ($\frac{{U_{D} }}{U}Rd\theta$) and an extra displacement (expressed as a fraction of the first) induced by the external force, $F_{L}$ in this case. In the first half of the channel, $F_{L}$ is directed towards the inner wall and $U_{{FL}}$ opposes $U_{D}$, translating into a lag that is sensitive to the particle size. Based on Eq. (8), three scenarios can be distinguished:

##### Hyper-dominance of the secondary flow

If $U_{D} \gg U_{{FL}}$, the contribution of $F_{L}$ to the displacements is negligible. The trajectories of the particles will be very close to those of the fluid elements and therefore little separation will be achieved.

##### Dominance of the lift force

If, on the contrary, $U_{{FL}} > U_{D}$, there will be no displacement towards the outer wall as $F_{L} > F_{D}$, which is the case described for particle focusing. Please note the forces and their respective induced velocities are simply related by a scalar ($U = \frac{F}{{3\pi \mu a}}$), as explained in the theory section. Thus, comparing velocities is similar to comparing forces and, in this work, where we aim at explaining the particle trajectories, the use of velocities is more practical.

##### Moderate dominance of the secondary flow

If $U_{D} > U_{{FL}}$, with both being of similar order of magnitude, the contribution of $U_{{FL}}$ to the displacements becomes relevant. The trajectories of the particles will depend on their size and clearly different from that of the fluid, thereby achieving separation.

Bringing the analytical expressions from Eqs. (5), (6) and (7) into Eq. (8):

9 $$dx = CU_{m} W^{2} \frac{\rho }{\mu }\frac{{f_{{U_{D} }} (x)}}{{f_{U} (x)}}\left( {1 + \frac{J}{{3\pi }}\frac{{a^{3} R}}{{CU_{m} W^{5} }}\frac{{f_{{FL}} (x)}}{{f_{{U_{D} }} (x)}}} \right)d\theta$$

Equation (9) describes the infinitesimal displacement of particles and can be integrated to obtain their trajectories. Note how the extra displacement induced by $F_{{FL}}$ is strongly dependent on the particle size. The dependence of the net displacement on $U_{m}$ is also worth noting, as in a given system most variables will be fixed but $U_{m}$ is proportional to $Q$ and can be modified during the experiments. Higher $Q$ makes particles migrate faster towards the outer wall.

#### Resolution of the system

The resolution of a system can be defined as the distance achieved in the separation of two particles with a given difference in size. Mathematically, it is reflected by the variation of the relative displacement with a variation in particle size:

10 $$\frac{{\partial \left( {\frac{{U_{{FL}} }}{{U_{D} }}} \right)}}{{\partial a}}\sim \frac{{a^{2} R}}{{C_{{ROI}} U_{m} W^{5} }}$$

It can be seen that the resolution will be higher for larger particles in a given system with fixed geometry. On the other hand, for a given set of particles, the resolution can be magnified, for instance, by a larger radius or a narrower channel.

#### Trajectories of particles within the separation section

The integration of Eq. (9) provides the position of a particle along the symmetry line as a function of the arc advanced through the curved microchannel. Given its complexity, it was solved by a finite element approach. Discrete displacements ($\Delta x$) along the symmetry line were calculated by introducing a finite value for $d\theta$ ($\Delta \theta$) in Eq. (9):

11 $$\Delta x = CU_{m} W^{2} \frac{{f_{{U_{D} }} (x)}}{{f_{U} (x)}}\left( {1 + \frac{J}{{3\pi }}\frac{{a^{3} R}}{{CU_{m} W^{5} }}\frac{{f_{{FL}} (x)}}{{f_{{U_{D} }} (x)}}} \right)\Delta \theta$$

Accordingly, the position of the particle can be obtained by the summation of the finite displacements:

12 $$\begin{array}{*{20}c} {x_{1} = x_{0} + \Delta x_{0} } \\ {x_{2} = x_{1} + \Delta x_{1} } \\ \vdots \\ {x_{n} = x_{0} + \mathop \sum \limits_{0}^{{n - 1}} \Delta x_{i} } \\ \end{array}$$

## Material and methods

### Device fabrication

The devices were fabricated on hybrid silicon-glass systems so they could stand up high pressures without undergoing deformation. First, the microchannels were patterned on a silicon wafer with photoresist 1813 (chromium mask; Micro Lithography Services Limited). The microchannels were dry etched with the photoresist as mask using a short cycle Bosch process to minimize the roughness of the sidewall (Tegal dry etcher; ~ 200 nm escalloping). The wafer was then cleaned and 0.5 µm of Al were sputtered to cover the channels and act as an etch stop for the next step. Lithography was done on the back side (plastic mask; Micro Lithography Services Limited) and via holes were dry etched for the fluidic connections (Tegal dry etcher; ~ 2 µm escalloping). With all the micromachining finished, the silicon wafer, together with a borosilicate wafer, was cleaned and activated in piranha solution for 15 min. Both wafers were put together and anodically bonded (380 °C and 1 kV for 4 h). Last, glass capillaries (Genetec, 100 and 170 µm inner and outer diameter, respectively) that served as fluid connections were glued with epoxy (EPO-TEK 302-3M).

The microchannels consisted of a focusing section comprised of two loops with $R$ 500 µm, followed by a separation section comprised of a half loop with $R$ 100, 200 or 300 µm. The dimensions were 6.15 × 14.5 µm ($W \times H$) (measured with CSI—ZYGO—3D Optical Profiler).

### Setup

Fluorescent polystyrene particles (0.79, 0.92 and 1.0 µm, Thermo Scientific Fluoro-Max) were suspended in deionized water (with 0.1% of Triton X to reduce agglomeration) in a concentration of ∼ 0.001 vol%.

An HPLC pump (Waters, model 515) was used to pump the samples through the devices at a controlled flow rate with a read out of the pressure.

During the operation, the devices were observed with an inverted fluorescence microscope (Olympus IX73 with an Orca-Flash 4.0 LT digital CMOS camera). Images were taken with a magnification of 20X and a 2 s exposure time.

### Simulations

Simulations of the fluid flow were performed in HARC microchannels using COMSOL Multiphysics v.5.5 (Laminar Flow interface; Navier–Stokes in a 3D space) in order to understand the main and secondary fluid flow ($U$ and $U_{D}$) at the symmetry line. The 3D flow was solved for water at room temperature in microchannels extending a quarter of a loop. The flow rate was set at one end (inlet) as fully developed flow, and the pressure was set to zero at the other end (outlet). The mesh generation was set to physics-controlled mesh and the maximum size of the elements was set to $W$/30. The main and secondary flow were analysed at a cross section ~ 2/3 of the channel length from the inlet to ensure a fully developed flow. With the results, analytical expressions were obtained for $U$ and $U_{D}$ at the symmetry line.

## Results and discussion

### Calculated trajectories

The finite displacements (Eq. 11) and position of the particle as a function of $\theta$ (Eq. 12) were solved for 0.79, 0.92 and 1.0 µm particles in microchannels with dimensions 6.15 × 14.5 µm ($W \times H$) and $R$ of 100, 200, 300 and 500 µm; values coincident with those in the experimental validation. The initial position of particles was set to $x_{0} = 0.25W$, and the finite angular displacement was $\Delta \theta = 2\pi /400$ to obtain a fine partition of the curved channels.

**Figure 3:**
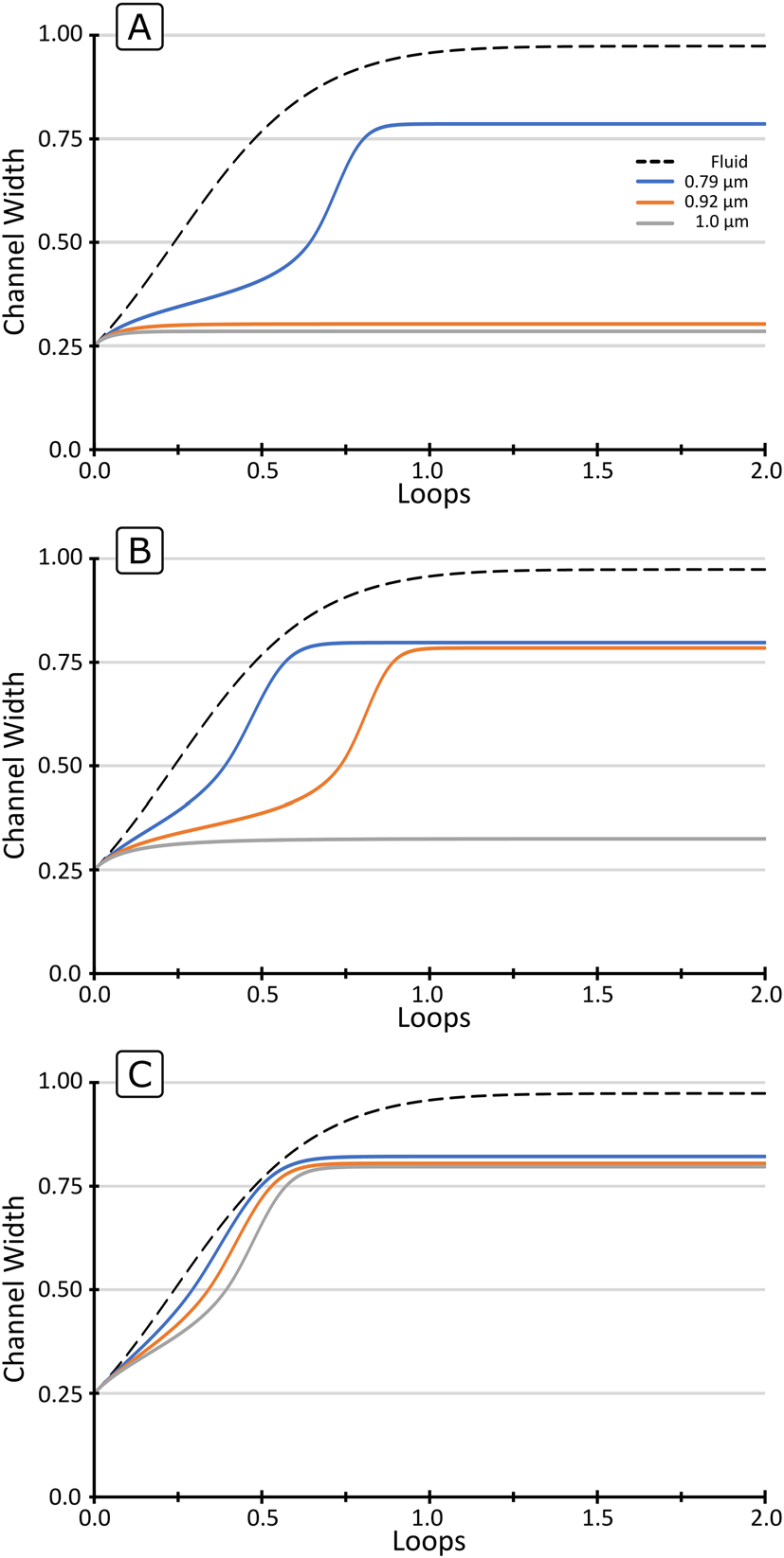
Trajectories of three particle sizes $a$ (0.79, 0.92 and 1.0 µm) in sections with fixed $Q$ (30 µL/min), $W$ (6.15 µm) and $H$ (14.5 µm). (**A**) $R$ 300 µm, (**B**) $R$ 200 µm and (**C**) $R$ 100 µm.

Figure 3 shows the trajectories of the three particle sizes $a$ (0.79, 0.92 and 1.0 µm) in microchannels with different $R$ (100, 200 and 300 µm) and fixed $Q$ (30 µL/min), $W$ (6.15 µm) and $H$ (14.5 µm). It can be seen how, when surpassing the Lift Barrier, particles migrate at different speeds towards the outer wall as the lag is proportional to $a^{3}$ (Eq. 9) and therefore follow different trajectories (Fig. 3B,C). In other cases, particles cannot surpass the Lift Barrier and remain at the inner wall, see Fig. 3A for 1 and 0.92 µm particles and Fig. 3B for 1 µm particles. At the same time, as discussed in Eq. (10), the resolution is heavily affected by $R$; compare the separation in Fig. 3A–C. For a too small $R$, $U_{D} \gg U_{{FL}}$ and the displacements are hyper-dominated by the secondary flow (Fig. 3C).

The weight of the lag becomes less and less relevant in the migration as $R$ is reduced and the trajectories of different particles converge to that of the fluid, resulting in little separation capabilities.

On the other hand, for a too large $R$, $U_{{FL}} > U_{D}$ and particles do not migrate (dominance of the lift force), also resulting in little separation capabilities for those particles remaining at the inner wall (Fig. 3A) for 0.92 and 1.0 µm particles. It can be concluded then that the separation section must fulfil $U_{D} > U_{{FL}}$ for the targets of interest, but both forces should be in the same range so the role of the lag is manifested (moderate dominance of the secondary flow), as represented in Fig. 3B.

**Figure 4:**
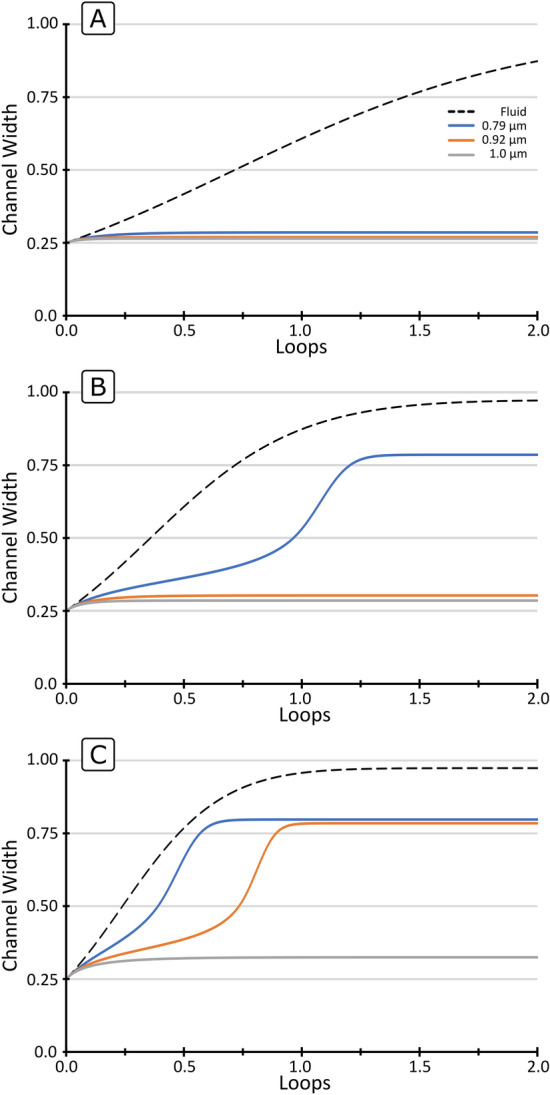
Trajectories of three particle sizes $a$ (0.79, 0.92 and 1.0 µm) in sections with fixed R (200 µm), $W$ (6.15 µm) and $H$ (14.5 µm). (**A**) $Q$ 10 µL/min, (**B**) $Q$ 20 µL/min and (**C**) $Q$ 30 µL/min.

Figure 4 shows the influence of $~Q$ (10, 20 and 30 µL/min) in the trajectories of the three particle sizes $a$. (0.79, 0.92 and 1.0 µm) in sections with $R$. (200 µm), $W$ (6.15 µm) and $H$ (14.5 µm), which reflects the influence of $U_{m}$ since $Q\sim U_{m}$. At the lowest $Q$, the particles are retained by the Lift Barrier, while at high enough $Q$, particles surpass it and migrate towards the outer wall. This effect originates in the fact that the strength of the secondary flow grows faster with $U_{m}$ than the migration induceby $F_{L}$ (Eqs. 6 and 7), which has also been reported in other studies^25,27,35,36^.

The balance can thus be shifted from lift-dominance (Fig. 4A), to secondary flow-dominance by the increase in $Q$ (Fig. 4B,C). On the other hand, the higher the $Q$, the faster particles migrate. It can also be seen that the migration is slower in the first half of the channel, where $F_{L}$ opposes the displacement, and it becomes faster in the second half, where $F_{L}$ adds up to the secondary flow.

**Figure 5:**
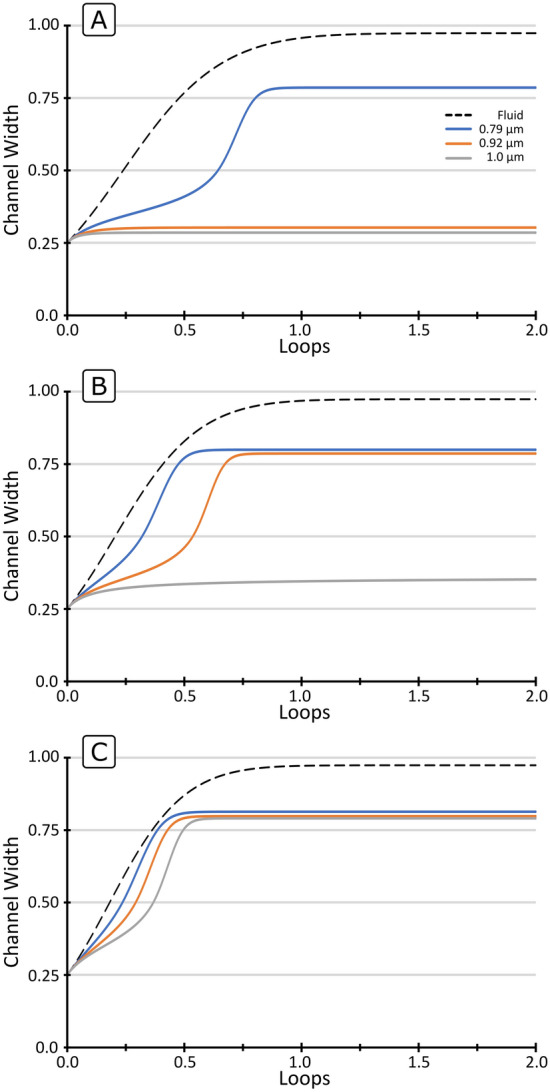
Trajectories of three particle sizes $a$ (0.79, 0.92 and 1.0 µm) in sections with fixed R (300 µm), $Q$ (30 µL/min) and $H$ (14.5 µm). (**A**) $W$ 6.15 µm, (**B**) $W$ 6.65 µm and (**C**) $W$ 7.15 µm.

Finally, Fig. 5 shows the influence of $W$ (6.15, 6.65 and 7.15 µm) in the trajectories of three particle sizes $a$ (0.79, 0.92 and 1.0 µm) in sections with a fixed $R$ (300 µm), $Q$ (30 µL/min) and $H$ (14.5 µm). Note that a change in $W$ while keeping $Q$ and $H$ fixed translates into a change in $U$ and $AR$ as well. Overall, widening the microchannel results in a shift in the balance of forces towards secondary flow dominance, in a similar trend to decreasing in $R$.

The dependence of the trajectories on the variables $a$, $R$, $Q$, and $W$ was illustrated individually to provide a notion of their effect on the separation. The variable $H$ also affects the migration; deeper channels favour the lift force dominance (Eq. 10), in a similar trend to increasing $R$. In a device, sections with different dominance can be placed in series and achieve the desired particle separation. For instance, a microchannel fulfilling $U_{{FL}} > U_{D}$ (lift force dominance) can first focus all particles close to the inner wall and to later change to $U_{D} > U_{{FL}}$ (secondary flow dominance) and allow particles to migrate and achieve their separation, as it will be shown in the experimental results. This change may be induced by a modification in $R$, $Q$, $W$ and $H$. However, for a microchip with a single layer and one channel, $H$ and $Q$ will be constant throughout the device. On the other hand, the dominance can easily be changed by a modification of $W$ or $R$ between the different sections of the microchannel.

As it was discussed, contrary to HARC systems for particle focusing, where the focus position is stable even if the parameters of the system suffer moderate changes, in HARC systems for particle separation every variable affects the performance. Therefore, for particle separation, HARC systems must be carefully designed, fabricated with small tolerances and operated with stable equipment.

### Experimental separation

The chips used for the experimental demonstration consisted of a focusing section followed by a separation section with a smaller $R$.

**Figure 6:**
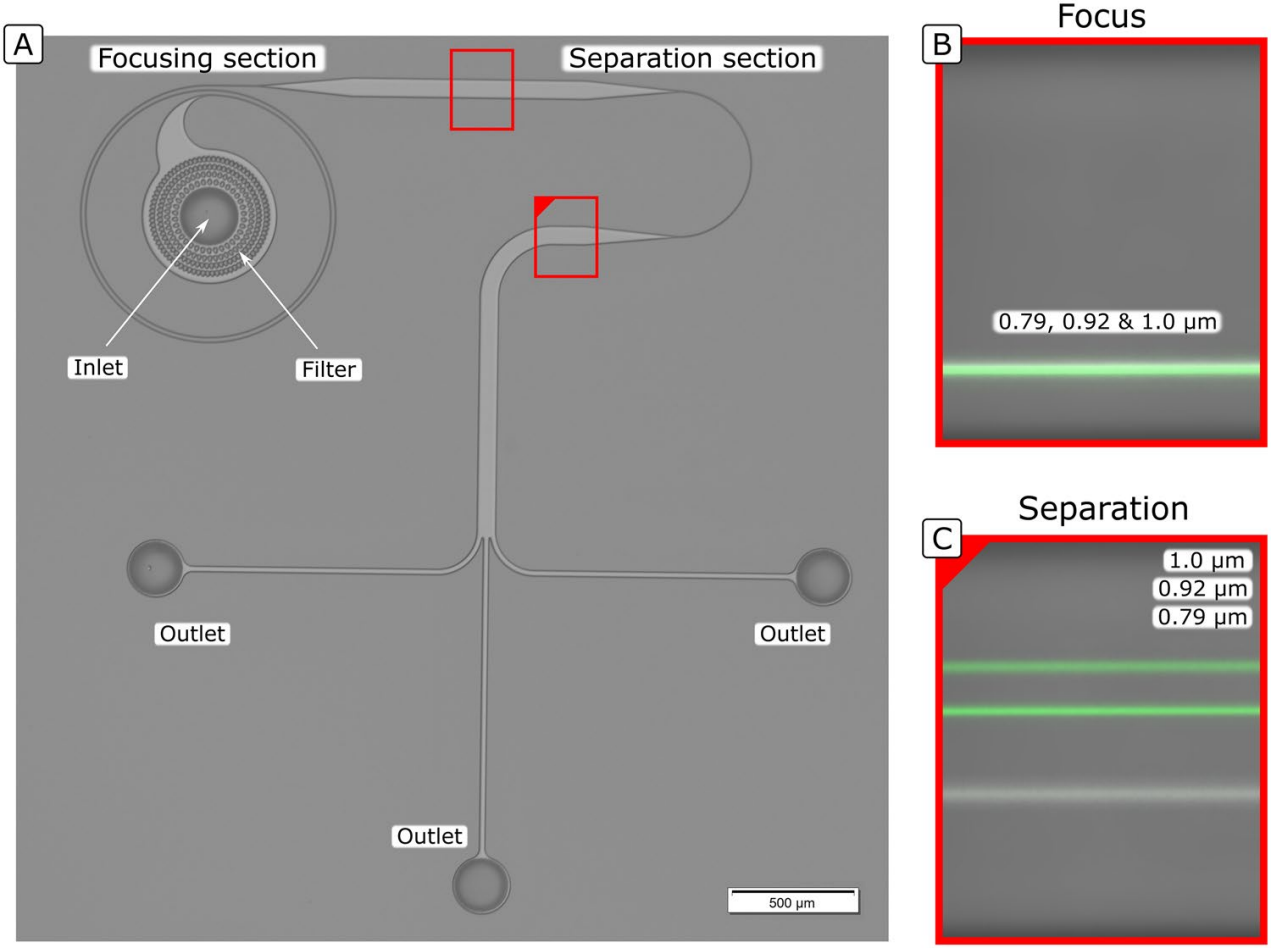
(**A**) View of HARC system under the microscope. (**B**) Example of performance of the focusing section (two loops, $R$ 500 µm, 6.15 × 14.5 µm ($W \times H$), $Q$ 32 µL/min). (**C**) Example of performance of the separation section (half loop, $R$ 200 µm, 6.15 × 14.5 µm ($W \times H$), $Q$ 32 µL/min).

A HARC system under the microscope can be seen in Fig. 6A, together with an example of focus and separation performance with 0.79, 0.92 and 1.0 µm particles, Fig. 6B,C, respectively. The focusing section consisted of two loops, $R$ 500 µm and 6.15 × 14.5 µm ($W \times H$, measured values) followed by 250 µm of straight segment to allow the particles to relax after the curve and initiate the separation from a closer position. The separation section consisted of half loop with similar cross section and smaller $R$ (100, 200 and 300 µm were evaluated). After each section, an expanded channel ($W$ 80 µm) was included to allow for a better visualization under the microscope.

**Figure 7:**
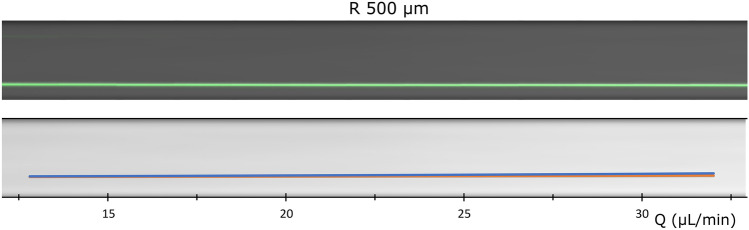
Performance of the focusing section (two loops with $R$ 500 µm followed by 250 µm of straight segment, 6.15 × 14.5 µm ($W \times H$)) with 0.79, 0.92 and 1.0 µm particles. Experimental (top) and prediction by the model (bottom).

Figure 7 shows the experimental performance of the focusing section—the observed position of the particles at the following expanded region as a function of the flow rate—together with the prediction of the model. Both results showed that the three particle sizes focus at the inner wall up to the flow rate allowed by the pressure limitation in our systems (~ 32 µL/min). In the model, the smallest particle (0.79 µm) had a slightly more advanced position towards the outer wall, especially at the highest flow rates. In the experiments, this was avoided by the short straight segment after the focusing section, that pushed particles back towards the inner wall. Regarding the minimum $Q$ to achieve focus, particles were expected to be focused at a single position over 24 µL/min (Eq. 1), extended as a plane for lower values, and poorly focused below 12 µL/min. Indeed, in the experiments, a faint trace was observed at the outer wall, which faded away for $Q$ higher than ~ 14 µL/min.

**Figure 8:**
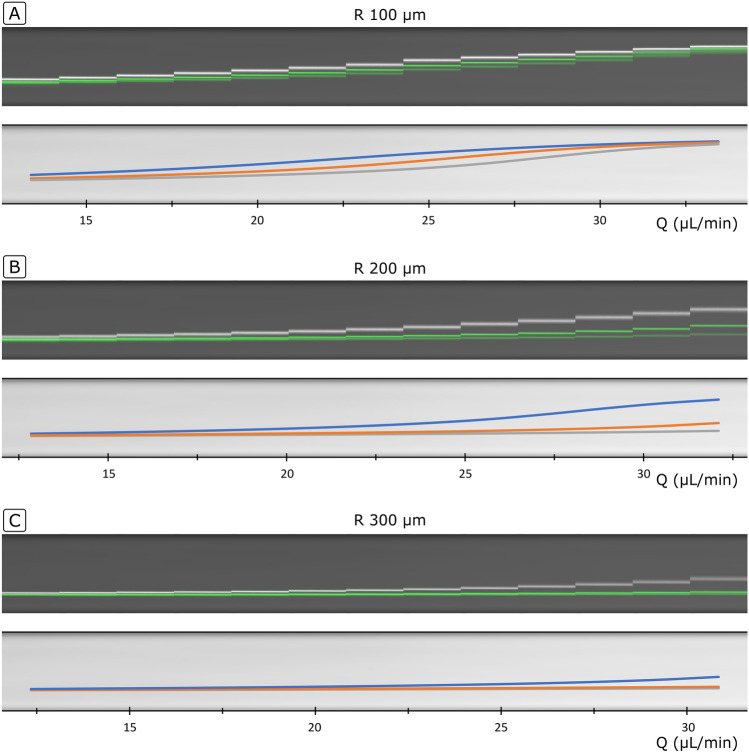
Performance of the separation section (half loop, 6.15 × 14.5 µm ($W \times H$)) with 0.79, 0.92 and 1.0 µm particles. Experimental positions observed at the 80 µm wide expansion of the channels (top) and prediction by the model (bottom). (**A**) $R$ 100 µm, (**B**) $R$ 200 µm, (**C**) $R$ 300 µm.

Figure 8 shows the experimental performance of the three separation sections ($R$ 100, 200 and 300 µm)—the observed position of the particles at the following expanded region as a function of the flow rate—together with the prediction by the model. It can be seen how, with a small enough $R$, which translates into $F_{D}$ surpassing $F_{L}$, particles do migrate towards the outer wall and follow different trajectories, thereby achieving their separation. In agreement with the discussion in the section “Calculated trajectories”, $Q$ and $R$ have a strong impact on the migration and resolution. In the three cases, at the lowest $Q$, particles were still close to the inner wall after the separation section. This is due to the fact that lower $Q$ favours a lift-dominance and results in a slower migration of the fluid towards the outer wall. As $Q$ was increased, the particles reached further towards the outer wall. On the other hand, the resolution of the system was strongly dependent on $R$. For a $R$ of 100 µm, the secondary flow had a dominant role and the trajectories were close to each other (Fig. 8A). The distance between the trajectories was amplified for $R$ 200 µm (Fig. 8B). Last, for $R$ 300, the resolution showed a similar trend, with 0.79 µm particles having an advanced migration at the highest $Q$ while 0.92 and 1.0 µm barely migrated (Fig. 8C). The limitation of 200 bar, however, impeded evaluation at higher $Q$.

It can also be observed that the resolution goes hand by hand with the flow rate. The distance between the trajectories was amplified with $R$, but the systems were longer and needed higher flow rates until particles started the migration, thereby demanding a higher pressure. In the range of sub-micron particles, the pressure demand is a critical parameter, as it quickly reaches the limit of the technology and sets a practical limit for the resolution.

The three particle sizes, with a difference in size down to 80 nm, were successfully focused and widely spread over the microchannel, verifying the feasibility of the proposed technology for particle separation with extremely high resolution. Potential applications for such a fine particle manipulation are, for instance, focusing and separation of bacteria by species or by size within a population, which may be of interest for studies of antibiotic resistance or cell synchronization. It can also be seen that the model, although developed with important simplifications, explains the trajectories remarkably well, capturing when the particles remain at the inner wall (focusing mode) and when they surpass the Lift Barrier, including their migration thereafter (separation mode). The agreement between the experiments and the model validates the hypothesis for the migration velocities and the discussion in the previous paragraphs in relation to the impact of the different variables. The trajectories of particles in the microchannels could be accurately described by assuming that they move with the same speed as the fluid and imposing a migration velocity induced by the lift force. The model being accurate also supports the recently proposed analytical formula for the strength of lift force^25^ and that its distribution ($f_{{FL}} (x)$) along the central line is close to a parabola, as already predicted by Ho & Leal in 1974^26,33^. On the other hand, the model can be improved, for instance, by a finer definition of $f_{{FL}} (x)$, as here it was simplified to a similar parabola for all particle sizes and $Re$ numbers. In the literature, small variations as function of particle size and $Re$ have been reported^33,34^. The initial position was also considered to be fixed at 0.25 $W$, while it may suffer subtle variations depending on the preceding section. Last, in this work the experimental positions were observed at an expanded outlet, which may induce minor modifications.

Although mathematically the separation resolution can reach a few nm, it must not be confused with the capacity to use the HARC systems to focus and separate particles with sizes of a few nm. To focus and separate nano-particles, much smaller microchannels are needed and, with the pressure to run the systems scaling with the square of the target size^12^, the capacity to manipulate such particles lies well beyond our limit of 200 bar. Nevertheless, in the systems presented here, ~ 90% of the pressure drops in the focusing section. The use of an alternative method for the pre-focusing may alleviate the pressure demand and therefore allow the application of HARC systems for the separation of much smaller particles.

Last, while HARC systems offer the means for focusing a range of particles in as stable position largely unaffected by small variations in the variables defining the system, when using HARC systems for particle separation, every variable contributes to the performance (see Eq. 9). For a tailored outcome, especially when targeting sub-micron particles, the tolerances in the fabrication must be exquisite and the pump capable of maintaining a very stable flow.

## Conclusions

Inertial focusing in High Aspect Ratio Curved systems (HARC systems) not only offers the possibility to focus randomly distributed particles of different sizes into a single position, but also allows for their separation by size with mathematically unlimited resolution (demonstrated down to 80 nm). The experiments agreed well with the proposed model, thereby enabling the precise engineering of the systems.

While the performance for particle focusing in HARC systems is not affected by moderate changes in the parameters of the system, a careful design, precise fabrication and stable operation are needed for particle separation, as the trajectories are very sensitive to multiple variables ($U$, $a$, $W$, $H$ and $R$).
